# Effects of edge cracks on the thermomagnetic instabilities of type-II superconducting thin films

**DOI:** 10.1093/nsr/nwad052

**Published:** 2023-02-28

**Authors:** Ze Jing

**Affiliations:** Institute of Extreme Mechanics and School of Aeronautics, Northwestern Polytechnical University, Xi’an 710072, China

**Keywords:** superconducting thin film, thermomagnetic instability, flux jumps, edge crack, scaling law

## Abstract

Thermomagnetic instability is a crucial issue for the application of superconductors. Effects of edge cracks on the thermomagnetic instability of superconducting thin films are systematically investigated in this work. Dendritic flux avalanches in thin films are well reproduced through electrodynamics simulations, and relevant physical mechanisms are revealed from dissipative vortex dynamics simulations. It is found that edge cracks sharply decrease the threshold field for the thermomagnetic instability of superconducting films. Spectrum analysis shows that the time series of magnetization jumping displays scale-invariance and follows a power law with an exponent around 1.9. In a cracked film, flux jumps more frequently with lower amplitudes compared with its crack-less counterpart. As the crack extends, the threshold field decreases, the jumping frequency gets lower, while its magnitude gets larger. When the crack has extended long enough, the threshold field increases to even larger than that of the crack-less film. This counterintuitive result originates from the transition of the thermomagnetic instability triggered at the crack tip to the one triggered at the center of the crack edges, which is validated by the multifractal spectrum of magnetization jumping sequences. In addition, with the variation of crack lengths, three different modes of vortex motion are found, which explains the different flux patterns formed in the avalanche process.

## Teaser

Structural defects dramatically change the stability of superconductivity: the threshold filed, avalanche patterns and multifractal spectrum of the thermomagnetic instability process transit with the extension of edge cracks in superconducting films.

## INTRODUCTION

For type-II superconducting (SC) thin films under an increasing magnetic field or self-field of a transport current, magnetic field penetrates the sample in the form of flux lines (also known as Abrikosov vortices) [1,2]. According to Bean's critical state model [3], the vortices will be statically pinned at pinning sites where the pinning force counterbalances the driving force, and the penetrated vortices are distributed nonuniformly within the superconductor (where vortex density is largest at the surface and decreases deep into the interior) [4,5]. However, this critical state is metastable. Any tiny disturbance will lead to vortex motion, which dissipates energy and destabilizes the critical state [6,7]. If the dissipated heat cannot be removed in a timely manner, the local temperature rise weakens pinning and leads to the motion of more vortices. Eventually, a positive feedback loop is formed, and thermomagnetic instability (TMI) develops in the superconductor [8].

TMI is usually considered unfavorable for the application of SC thin films, which degrades the current carrying capacity due to the suppression of superconductivity in the hotspot area [9], introduces electromagnetic noises to SC devices during sudden entry or exit of vortices [10], and induces thermal strain or even damage to the material [11,12] due to the sharp temperature rises at hotspots. On the other hand, it was reported that TMI can be utilized to design flux injectors [13] or to magnetize bulk superconductors [14]. Experimental observations [15,16] and numerical simulations based on the cellular automaton [17] show that the distribution of vortex avalanche sizes, the duration and temporal power spectra follow power laws and demonstrate finite-size scaling which are indications of self-organized criticality (SOC) [18]. More interestingly, direct magneto-optical imaging (MOI) observations revealed dendritic flux avalanche patterns in SC thin films during the TMI events [8,19]. Numerical simulations and analytical analysis of the coupled electromagnetic and heat diffusion equations show that the dendritic flux avalanches result from the branching of the propagating flux lines due to the positive feedback between the nonlocal flux motion and Joule heating [20–22]. Experimental measurements using MOI and linear stability analysis led to a consensus that a threshold field, ${\mu }_0{H}_{{\rm{th}}}$, exists for the onset of dendritic flux avalanches [23,24]. Hence, vortex dynamics and the dissipative vortex motion induced TMI in SC films are of both practical importance to the application of SC devices and theoretical interest for studies of non-equilibrium dynamics of the complex system of vortices.

Recent MOI observations and numerical simulations have revealed that random or structured defects significantly affect the TMI behavior of SC thin films [25–30]. Within inhomogeneous SC films patterned with arrays of antidots [25,26,28], blind holes [27,29] and insulating particles [30], it is found that inhomogeneities strongly modify the current carrying capacity and dramatically change the flux avalanche patterns. Quantitative MOI measurements of the vortex avalanches in SC films show that the probability distributions of avalanche sizes follow power laws and demonstrate finite-size scaling [31,32]. In a recently-developed hypervelocity MOI system with two branches of pulsed laser, it is found that optically triggered vortex avalanches are driven by magnetic pressure and showed SOC and chaotic dynamics different from that commonly found [33]. Further investigations show that structural defects such as edge slits [34], cracks [35], and indentations [36] can dramatically change the TMI of SC films, and from where dendritic flux avalanches can be triggered. It was also reported that field-dependence of the critical current density ${J}_c$ plays a crucial role in flux avalanches and the reduction of ${J}_c$ by large local magnetic fields at a crack tip can be tuned to selectively trigger TMI [37]. However, it lacks quantitative investigation for the effects of structured defects on the TMI of SC thin films, especially detailed statistics on the avalanche dynamics of structured SC thin films.

In this work, the effects of edge cracks on the TMI of SC thin films subjected to an external magnetic field are investigated. The effects of edge cracks on the threshold condition for the onset of TMI, the dendritic flux avalanche patterns as well as the scaling laws and the multifractal spectrum of SC films are systematically investigated. Moreover, the physical mechanisms for the transition of vortex patterns in the cracked SC thin films during TMI events are revealed.

## RESULTS AND DISCUSSION

### Dendritic flux avalanches in the crack-less thin film

**Figure 1.**
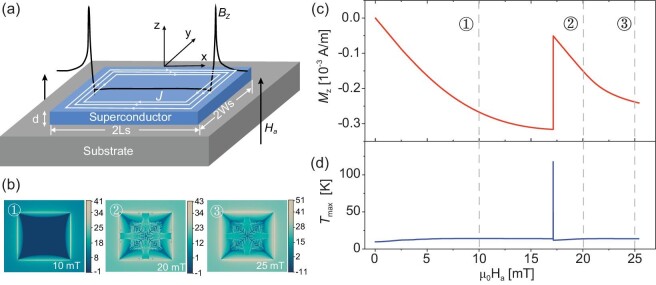
(a) Schematic diagram of the SC thin film subjected to a magnetic field ${H}_a$ which is perpendicular to the film plane. (b) Dendritic flux avalanche patterns in the SC thin film when the field increased from zero to 25 mT with a ramp rate of 50 T/s, at the ambient temperature *T*_0_ = *T_c_*/4. (c) Average magnetization and (d) maximum temperature of the SC film during the flux avalanche process.

As illustrated in Fig. 1(a), a rectangular SC thin film of length $2{{\rm{L}}}_{\rm{s}}$, width $2{{\rm{W}}}_{\rm{s}}$, and thickness ${\rm{d}}$ is investigated. The film is initially zero-field-cooled to the ambient temperature ${T}_0$ (below the critical temperature ${T}_c$), thermally in contact with the substrate, and exposed to an increasing magnetic field with a ramp rate of ${\mu }_0{\dot{H}}_a$. The relationship between the electric field ${\boldsymbol{E}}$ and the current density ${\boldsymbol{j}}$ of superconductors [1,38] is assumed to follow the nonlinear E–J power law: ${\boldsymbol{E}} = {\rho }_f( j )\!{\boldsymbol{j}},$ with ${\rho }_f(j) = {\rho }_0{( {j/{j}_c} )}^{n - 1}$ for $j \le {j}_c,\ T \le {T}_c$, and ${\rho }_f(j) = {\rho }_{\rm normal}$ when $j > {j}_c\ or\ T > {T}_c$, where ${{n}}$ is the flux creep exponent which is temperature dependent and assumed to be $n = {n}_0( {{T}_c/T} )$, ${\rho }_0$ is a resistivity constant for the flux creep, and ${\rho }_{\rm nornmal}{\rm{\ }}$is the normal state resistivity, and ${j}_c$ is the critical current density which is a function of magnetic inductance ${\boldsymbol{B}}$ and local temperature *T*. Here, a linear temperature dependence and Kim's model [2] for the magnetic field dependence is assumed: ${j}_c = {j}_{c0}( {1 - T/{T}_c} )/( {1 + | {\boldsymbol{B}} |/{B}_0} )$, in which ${j}_{c0}$ and ${B}_0$ are material dependent parameters. The material parameters are assumed to be the typical parameters of MgB_2_ [39] with ${\rho }_0 = {\rho }_{\rm normal} = 7\ \times {10}^{ - 8}\ {\rm{\Omega }} \cdot {\rm{m}}$, ${T}_c = 39\ {\rm{K}}$, ${n}_0 = 20$, ${j}_{c0} = 1.2\ \times {10}^{11}\ {\rm{A}}/{{\rm{m}}}^2$, and ${B}_0 = 50\ {\rm{mT}}$. The heat capacity is assumed to be ${{{C}}}_p = 35{( {T/{T}_c} )}^3\ {\rm{kJ\ }}{{\rm{m}}}^{ - 3}{\rm{\ }}{{\rm{K}}}^{ - 1}$, the thermal conductivity ${\rm{\kappa \ }} = {\rm{\ }}170{( {T/{T}_c} )}^3\ {\rm{W\ }}{{\rm{m}}}^{ - 1}{\rm{\ }}{{\rm{K}}}^{ - 1}$, and the heat transfer coefficient between the SC film and the substrate ${{h\ }} = {\rm{\ }}220{( {T/{T}_c} )}^3\ {\rm{kW\ }}{{\rm{m}}}^{ - 2}{\rm{\ }}{{\rm{K}}}^{ - 1}$.

To verify the modelling scheme and to reveal the effects of the edge crack on the TMIs of superconductors, the TMI of a square-shaped crack-less SC film of length and width $2{{{L}}}_{\rm{s}} = 2{{{W}}}_{\rm{s}}$= 4.4 mm, and thickness ${{d\ }} = {\rm{\ }}0.5{\rm{\ \mu m}}$ is demonstrated and discussed first. Figure 1(b)–(d) show the flux patterns, the jump in the average magnetization and the maximum temperature of the film exposed to a magnetic field which is ramped from zero to ∼$25{\rm{\ mT}}$ with a ramp rate of $50{\rm{\ T}}/{\rm{s}}$ at ${T}_0 = {T}_c/4$. As demonstrated in Fig. 1(b), the magnetic flux smoothly penetrates the film from the edges until the external field reaches a certain threshold (∼17 mT). Then, the magnetic flux penetrates the film in the form of dendritic avalanches from the center of the edges (see images ② and ③ of Fig. 1(b)). This dramatic event changes the overall magnetization of the film and dissipates lots of energy in a short time, which manifests as the sharp jump in the average magnetization and maximum temperature as shown in Fig. 1(c) and (d). It can be seen from Fig. 1(c) that the maximum temperature rises much higher than the critical temperature.

To have a quantitative understanding on the criticality of TMI in superconductors, the threshold magnetic field, ${\mu }_0{H}_{th}$, i.e. the field when the first avalanche event occurs is investigated. As derived from perturbation analysis in Refs [40,41], at a small magnetic field, the TMI is mainly suppressed by lateral heat diffusion. Thus, the threshold field $H_{th}^k = \frac{{d{j}_c}}{\pi }{( {\frac{{{\pi }^2\kappa {T}^{\rm{*}}}}{{{\mu }_0{{\dot{H}}}_a{W}_s^3n{j}_c}}} )}^{1/5}$, in which ${T}^{\rm{*}} = {| {\partial ln{j}_c/\partial T} |}^{ - 1}$ and *w* is the half-width of the SC film. As the magnetic field penetrates deeper, the main mechanism for the suppression of TMI is heat removal, the threshold becomes $H_{th}^h = \frac{{d{j}_c}}{\pi }atanh[ {\frac{{h{T}^{\rm{*}}}}{{{\mu }_0{{\dot{H}}}_a{W}_sn{j}_cd}}} ]$. Under the adiabatic condition, the TMI is mainly suppressed by the heat capacity of superconductors, and the threshold field is given as $H_{th}^c = \sqrt {\frac{2}{\pi }\frac{d}{{{W}_s}}\frac{{{C}_p{T}^{\rm{*}}}}{{{\mu }_0}}} $.

**Figure 2.**
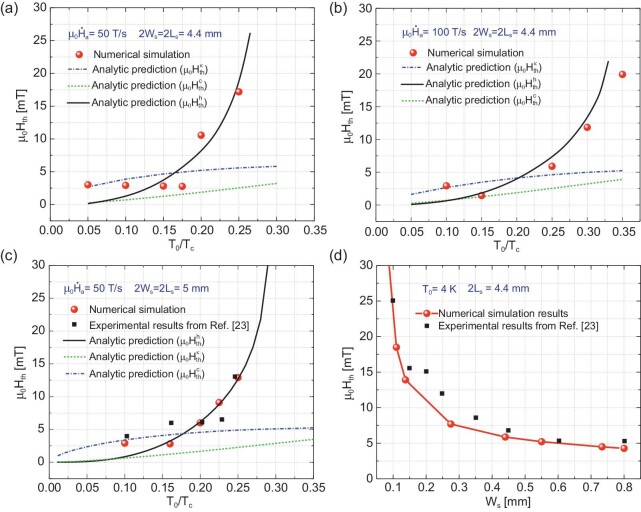
Threshold magnetic field for the onset of the dendritic flux avalanches in MgB_2_ thin film; (a) and (b) are the threshold field for the SC film of size 4.4 mm × 4.4 mm at different ambient temperatures with the magnetic field ramp rate of ${\mu }_0{\dot{H}}_a = 50\ {\rm{T}}/{\rm{s}}$ and 10$0\ {\rm{T}}/{\rm{s}}$, respectively, and (c) is for a SC thin film of size 5 mm × 5 mm at the ramp rate of ${\mu }_0{\dot{H}}_a = 50\ {\rm{T}}/{\rm{s}}$; (d) shows the variation of the threshold magnetic field with the half-width of the SC film at ${T}_0 = 4{\rm{\ K}}$.

The threshold fields of the crack-less SC film at different ${T}_0$ under the field ramp rate of ${\mu }_0{\dot{H}}_a\ $= 50 and 100 T/s are illustrated in Fig. 2(a) and (b), respectively. In the figures, the discrete red dots indicate the numerically simulated threshold magnetic fields. While, the blue-dash-dotted line, the green-dotted line and the black-solid line indicates the analytical threshold magnetic field $H_{th}^k$, $H_{th}^c$ and $H_{th}^h$, respectively (see online supplementary material for a colour version of this figure). As shown in Fig. 2, the threshold magnetic field exponentially increases with ${T}_0$. When the ambient temperature and the magnetic field are low, the simulated threshold field is close to $H_{th}^k$. While the ambient temperature gets higher, the simulated threshold magnetic field matches well with $H_{th}^h$. This is consistent with the assumptions that one takes when deriving the analytical expressions of the threshold field. Comparing the threshold values shown in Fig. 2(a) and (b), one can see that increasing the magnetic field ramp rate will lower the thermomagnetic stability of SC thin films. In addition, geometrical effects on the TMI are investigated. Figure 2(c) presents the threshold field ${H}_{th}$ of a 5 mm × 5 mm SC film at different ${T}_0$ with ${\mu }_0{\dot{H}}_a = 50\ {\rm{T}}/{\rm{s}}$, in which the black-squares indicate the experimental data from Ref. [23]. It shows that the simulation results are in good agreement with the experiments. Comparing Fig. 2(c) and (a), it can be inferred that the threshold field of TMI decreases when increasing the film sizes. Figure 2(d) shows the threshold field ${H}_{th}$ of a SC film of different aspect ratios, at 4 K and with ${\mu }_0{\dot{H}}_a = 50\ {\rm{T}}/{\rm{s}}$. The simulations also show good agreement with the experiments, and both results demonstrate that the threshold magnetic field exponentially decreases as the width of the SC film increases. All these results show that the numerical scheme developed reproduces well the essential characteristics of TMI in SC thin films.

### Effects of the edge crack on dendritic flux avalanches

**Figure 3.**
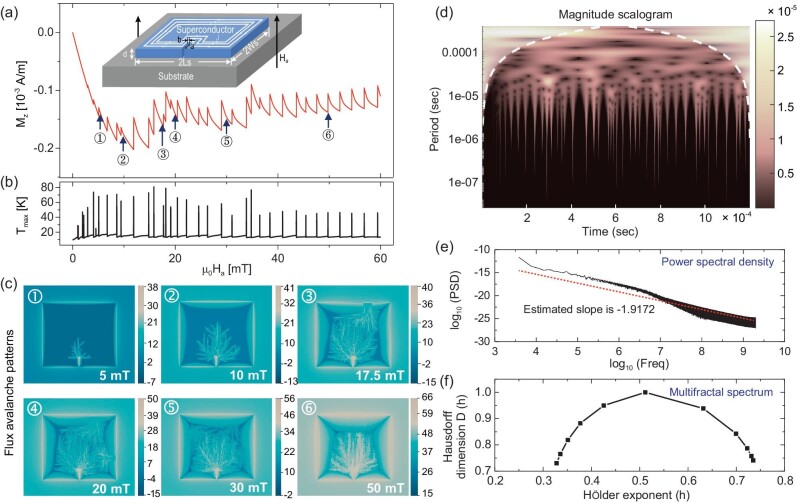
Dendritic flux avalanche behaviors of the SC thin film with an edge crack under an increasing magnetic field ramped from zero with a rate of 50 T/s at *T*_0_ = *T_c_*/4; (a) and (b) are the average magnetization and maximum temperature jumping curves, respectively; (c) shows the evolution of flux patterns during the avalanche process; (d) is the magnitude scalogram, which is the absolute value of the continuous wavelet transform of the magnetization jumping sequences, plotted as a function of time; (e) and (f) are the power spectral density and multifractal spectrum of the magnetization jumping sequences, respectively.

To reveal the effects of structural defects on the TMI, the coupled electromagnetic-thermal behaviors of a square-shaped SC thin film with an edge crack of length ${\rm{a}}$ and width ${\rm{b}}$ are investigated in this part. The geometry and material parameters of the film are the same as that of the crack-less film. Figure 3 shows the essential characteristics of the TMI behaviors in a SC thin film with a $0.66{\rm{\ mm}} \times 16.5{\rm{\ }}\mu {\rm{m}}$ edge crack (i.e. ${\rm{a}}/2{{\rm{W}}}_{\rm{s}} = 0.15$). As shown in Fig. 3(a) and (b), the magnetization and maximum temperature of the cracked thin film jump as the external field increases. Comparing with the jumping curves of the crack-less film, jumping frequency of the cracked film gets higher, which means that the SC film gets less stable due to the presence of the edge crack, while the jumping magnitude gets lower. Figure 3(c) displays the avalanche patterns at the moments indicated by arrows in Fig. 3(a). It demonstrates that dendritic avalanches initiate from the crack tip, and, as the field increases, it grows like a tree both in size and branches. When the main branch of this dendritic tree penetrates deep inside, new dendrites will be triggered from the opposite edge of the film, as image ③ of Fig. 3(c) shows. If the field further increases, more dendritic flux avalanches penetrate the film from other edges (see images ④ and ⑤ in Fig. 3(c)). When the field gets high enough, no new dendritic avalanche happens. And the dendrites, except for the one that nucleates from the crack tip, will be submerged by the penetrated magnetic flux.

Not only the topology of flux avalanche patterns manifests multiscale fractal features, the temporal evolution of the magnetic flux and maximum temperature is intermittent, with occasional bursts of various sizes which exhibit nonlinear power law behavior and multifractal characteristics that depend on higher-order moments and scales. Multifractal analysis is a method designed to understand the nonlinear nature of these time series across different time scales. For the avalanche signals with features occurring at different scales, multifractal analysis based on the wavelet method is conducted to elucidate the multiscale characteristics [18,42,43]. Figure 3(d)–(f) presents the multiscale temporal characteristics of the TMI events. For an intermittent signal, the magnitude scalogram, which is the absolute value of the continuous wavelet transform (CWT) of the signal, plotted as a function of time and frequency, can be more useful than the spectrogram [42]. The scalogram of the time series of magnetization jumping (Fig. 3(d)) captures the avalanche events at the time they occur, which reveals that there are low-frequency steady state oscillations in the magnetization from a larger time scale of ∼0.1 ms. As shown in Fig. 3(e), the power spectrum density (PSD) [43,44] of the time series of magnetization jumping (which indicates the distribution of avalanche sizes) shows a power law [8,15–18] with an estimated exponent of 1.9172, which demonstrates that the avalanche events are scale-invariant in time [42]. The multifractal spectrum [42,45], which shows the distribution of scaling exponents and provides a measure of the local regularity of the flux avalanche process, is shown in Fig. 3(f). The spectrum is broad with a wide-range of scaling exponents, which suggests that the avalanche process is multifractal.

**Figure 4.**
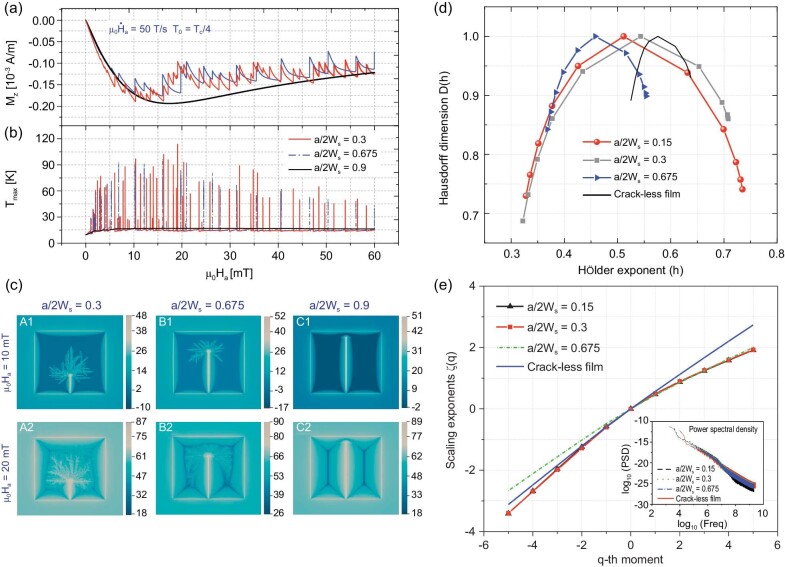
(a) and (b) Average magnetization and the maximum temperature jumping curves of the SC film with an edge crack of different lengths during the field increasing process. (c) Flux avalanche patterns of the SC film with different lengths of an edge crack. Images A1 and A2, B1 and B2 and C1 and C2 are the flux avalanche patterns in the films with a crack of length ${\rm{a}}/2{{\rm{W}}}_{\rm{s}} = 0.3,{\rm{\ }}0.675$ and 0.9, respectively, when the magnetic field reaches 10 mT and $20{\rm{\ mT}}$ at the ramp rate of $50{\rm{\ T}}/{\rm{s}}$, and ${T}_0 = {T}_c/4$. (d) Multifractal spectrum and (e) scaling exponents of the magnetization jumping signals. The inset image of (e) shows the power spectral density of the magnetization jumping sequences.

Additionally, to quantitively evaluate the effects of cracking on TMI, flux avalanches of SC thin films with various sizes of cracks are numerically simulated. In Fig. 4, flux dynamics of the cracked films under an increasing magnetic field ramped with a rate of 50 T/s at ${T}_0 = {T}_c/4$ is presented. Figure 4(a) and (b) shows the magnetization and maximum temperature of the SC films with an edge crack of lengths ${\rm{a}}/2{{\rm{W}}}_{\rm{s}} = 0.3$, 0.675 and 0.9, respectively. Like the cracked film with a crack length of ${\rm{a}}/2{{\rm{W}}}_{\rm{s}} = 0.15$, the SC films with an edge crack of lengths ${\rm{a}}/2{{\rm{W}}}_{\rm{s}} = 0.3$ and 0.675 also become unstable under a relatively low magnetic field. However, what is unexpected is that when the crack extends to almost split the film into two parts (such as ${\rm{a}}/2{{\rm{W}}}_{\rm{s}} = 0.9$), the film will never get into the avalanche state, which is proven by the smooth magnetization and maximum temperature curves. Figure 4(c) displays the flux patterns of the SC film that contains different lengths of edge cracks. For a small crack, flux avalanches nucleate from the tip of the crack and penetrates forward to the center of the film as the external field increases (see images A1 and A2). However, when the crack extends over half-width of the film, the avalanches initially nucleate at the crack tip, but the subsequently penetrated flux will trace back to the central area as the external field further increases (see image B2). Consistent with the magnetization and maximum temperature curves, magnetic flux smoothly penetrates into the cracked film with an edge crack of length ${\rm{a}}/2{{\rm{W}}}_{\rm{s}} = 0.9$ (see images C1 and C2). Thus, it can be concluded that the edge crack adds a new dimension to the TMI of SC thin films. Under the same external magnetic field and ambient temperature, modes of the TMI behaviors of the cracked SC thin films transit with the extension of the crack. The multifractal spectra (Fig. 4(d)) and the scaling exponents (Fig. 4(e)) for the flux avalanche process of the cracked SC film with various lengths of edge cracks further validate this transition. The support of the multifractal spectrum of the cracked film significantly expands compared with the narrow support of the monofractal spectrum of the crack-less film. In addition, support of the multifractal spectrum of the cracked film shrinks when the crack length extends over half-width. The scaling exponents also support this finding: as a function of the moments, the scaling exponents of the cracked film show a departure from linearity; while the crack extends over half-width it tends back to linear.

**Figure 5.**
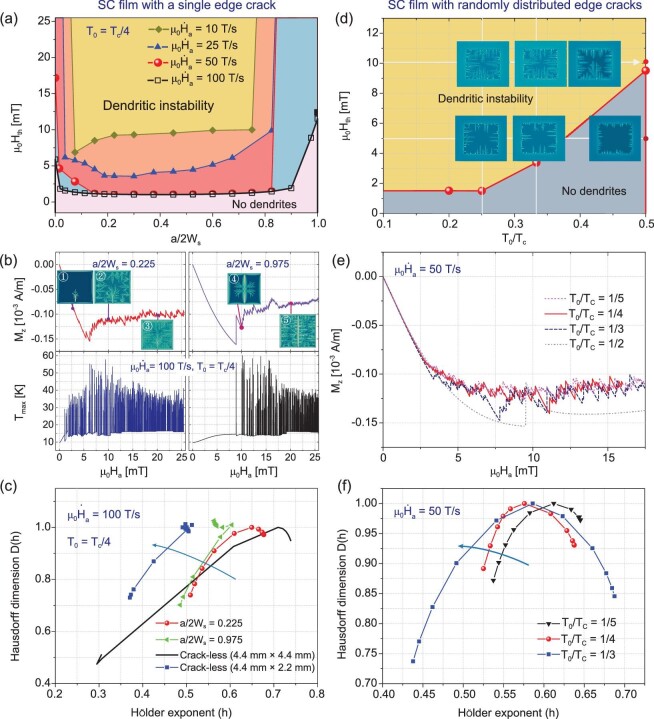
(a) Threshold magnetic field for the thermomagnetic instability of the SC film with different lengths of edge cracks. (b) Average magnetization and maximum temperature of the SC film with an edge crack of lengths ${\rm{a}}/2{{\rm{W}}}_{\rm{s}} = 0.225$ and 0.975, respectively, and (c) multifractal spectrum of the magnetization jumping process of the SC film subjected to an increasing magnetic field with a ramp rate of 100 T/s at ${T}_0 = {T}_c/4.$ (d) Average magnetization jumping. (e) Threshold magnetic field and (f) multifractal spectrum of the flux avalanche process for an SC film with randomly distributed edge cracks, subjected to an increasing magnetic field with a ramp rate of 50 T/s at ${T}_0 = {T}_c/4.$

In Fig. 5(a), the threshold magnetic fields as a function of the crack length at different field ramp rates are presented. It is shown that an edge crack sharply decreases the threshold field of the SC film. Under an increasing field with a ramp rate of 25 T/s and at ${T}_0 = {T}_c/4$, the crack-less film will never enter into the thermomagnetic instability state. However, a small crack of length ${\rm{a}}/2{{\rm{W}}}_{\rm{s}} = 0.0375$ makes it unstable when the external magnetic field reaches 6.5 mT, as indicated by the steep drop of the threshold magnetic field curve in the TMI phase diagram. If the crack length further increases the threshold magnetic field first decreases and then sharply increases as the crack length approaches the width of the film. For the SC film with a crack length larger than ${\rm{a}}/2{{\rm{W}}}_{\rm{s}} = 0.825$, it will never enter into the thermomagnetic instability state when exposed to an increasing magnetic field with a ramp rate of 25 T/s and 50 T/s at ${T}_0 = {T}_c/4$.

Under an increasing magnetic field, with a much larger ramp rate of 100 T/s, the threshold magnetic field sharply increases to a value even larger than the crack-less film as the crack extends to the opposite edge of the film. This seems unexpected but reasonable: when the crack extends to split the film into two parts, the threshold magnetic field should be close to that of the crack-less film with half-width. Under an increasing field with ramp rate 100 T/s and at ${T}_0 = {T}_c/4$, the threshold field of a crack-less film with width $2{{\rm{W}}}_{\rm{s}} = 2.2\ {\rm{mm}}$ is $11.24\ {\rm{mT}}$, which is close to the value indicated by the black square. In Fig. 5(b), the average magnetization, the maximum temperature and the corresponding flux avalanche patterns of the SC films with an edge crack of lengths ${\rm{a}}/2{{\rm{W}}}_{\rm{s}} = 0.225$ and 0.975 are presented. As demonstrated in Fig. 5(b), the average magnetization of the SC film with a small crack, jumps with small magnitudes early on as the field increasing process develops. For the film with an extended crack, the magnetization jumps at a higher field and with larger magnitudes. Flux avalanches nucleate from the crack tip for the SC film with a small crack (see the inset image ①), while they nucleate near the central area of the crack edges for the film with an extended crack (see the inset image ④). In addition, different from the case under external field with a lower ramp rate, support of the multifractal spectrum of the magnetization jumping sequences shrinks as the crack extends when the SC film is subjected to a quickly ramping field (Fig. 5(c)).

To further illustrate the effects of cracks on the TMI of SC films in practical applications, the flux avalanche behaviors of a cracked film with 10 randomly distributed edge cracks (with $0.015 < {\rm{a}}/2{{\rm{W}}}_{\rm{s}} < 0.045$) on each side are presented in Fig. 5(d)–(f). Compared with crack-less film (Fig. 2(a)), threshold magnetic field of the cracked film (Fig. 5(d)) significantly decreases. It is also seen that the TMI thresholds of SC films with multiple cracks increase as the ambient temperature increases. Even at ${T}_0/{T}_c = 0.5$, the cracked film can get into TMI state. Due to the multiple cracks, the flux jumps more frequently with smaller magnitudes compared with that of the film with a single crack (Fig. 5(e)). Moreover, seen from the multifractal spectra of the SC film with multiple cracks under different ambient temperatures shown in Fig. 5(f), support of the multifractal spectrum expands as the ambient temperature increases.

### Mechanisms of dendritic avalanches of cracked SC film

As discussed above, the edge crack is one of the driving factors that influences the TMI of SC thin films. The dendritic flux avalanche patterns transit with variation of the crack lengths. Under certain conditions, cracks even determine whether a SC film can be unstable. In this section, the physical mechanism of the dendritic flux avalanches is discussed from the perspective of vortex dynamics. Although the length scales in time-dependent Ginzburg-Landau (TDGL) theory are different from the macroscopic flux dynamics and the results are not directly comparable with the macroscopic simulations, TDGL simulations which couple the heat diffusion with vortex motion are the best, currently available, computational tool to qualitatively simulate the dissipative vortex dynamics and elucidate the formation of flux patterns in the squared SC thin film with different lengths of edge cracks.

**Figure 6.**
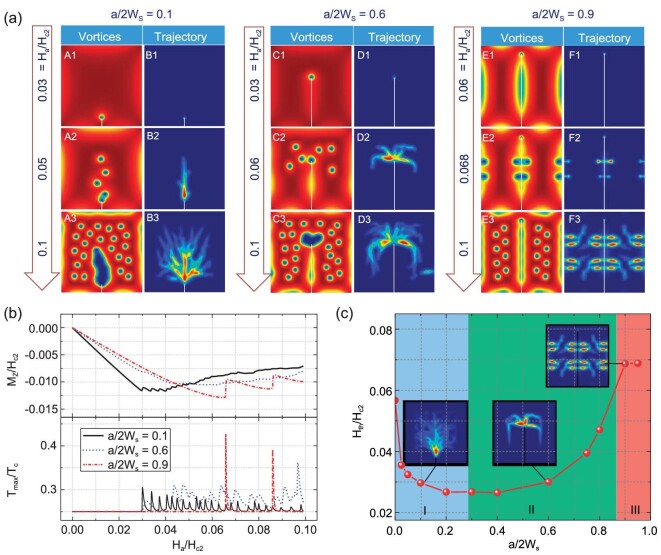
TDGL simulations of the vortex dynamics of a 60 × 60 SC film with an edge crack of lengths a/2Ws = 0.1, 0.6 and 0.9. (a) Distribution of the Cooper pair density and the trajectories of the vortices in the film. (b) Average magnetization and maximum temperature of the film during the field increasing process. (c) Threshold magnetic field when the first vortex penetrates the film, and the inset images demonstrate different vortex penetration patterns.

Figure 6(a) shows the simulated Cooper pair density distribution and trajectories of vortices during the field increasing process for a 60 × 60 SC film with an edge crack of lengths a/2Ws = 0.1, 0.6 and 0.9. As seen from image A1 of Fig. 6(a), initially, vortices penetrate the film from the crack tip when the crack is small. If the crack extends long enough (0.9 times the film width here), the vortices penetrate the SC film from the center of the crack edges (see images E2 and F2 of Fig. 6(a)). When the magnetic field further increases, more vortices penetrate the film. However, the penetration paths of the vortices (i.e. vortex trajectories) manifest different modes in the film with various lengths. For the film with a small edge crack, the initially penetrated vortices form a straight path. As the field increases, more vortices penetrate the film and are repelling with each other, which leads to the so-called crowding effect and the branching in vortex trajectories. Although it is qualitative, the branched vortex trajectories shown in images B2 and B3 of Fig. 6(a) resemble the flux patterns in images A1 and A2 of Fig. 4(c). If the crack extends to an intermediate length, the vortices also penetrate into the film from the crack tip. However, the vortices will trace back to the middle of the cracked film as the field further increases (see images C2–C3 and D2–D3 in Fig. 6(a)). The vortex trajectories in the film with an intermediate length of edge crack are quite similar to the flux patterns shown in images B1–B2 of Fig. 4(c). When the crack extends long enough, vortices initially penetrate the film through edges of the crack. As the external field increase further, more vortices will penetrate mainly from the side edges of the film parallel to the crack. Also, it can obviously be seen that the vortex trajectories (see images F2–F3 of Fig. 6(a)) of the film with long cracks resembles that of the flux avalanche patterns shown in inset images ④–⑤ of Fig. 5(c). Thus, it can be concluded that the transition of flux avalanche patterns in a cracked film originates from the change in the vortex motion paths.

Figure 6(b) shows the average magnetization and maximum temperature in the film as the field increases. It is seen that the jumping frequency of the average magnetization and maximum temperature gets lowered as the crack extends, however the magnitude significantly increases. This result is consistent with the findings presented in Fig. 4(a) and (b) and Fig. 5(b), which may have potential use in the application of SC thin films. If one finds the frequency of flux jumps decreases and its magnitude significantly increases, the SC film may have experienced significant structural damage. Moreover, the threshold magnetic fields when the first vortex penetrates into the film with cracks of different lengths are presented in Fig. 6(c). The simulated results reproduce well the trend found in the flux dynamics simulations. As it is shown, the threshold field sharply decreases due to the presence of the crack (region I). The threshold field increases as the edge crack extends to the opposite edge of the film (region II). When the edge crack extends long enough to almost split the film into two parts, the threshold field will keep constant. Therefore, it can be concluded that three modes of flux dynamics happen in the SC film as the edge crack continuously extends, which determines the thermomagnetic instability of the film.

## CONCLUSIONS

Structural defects introduced in the manufacturing or application process play significant roles in the TMI of SC thin films. This work focuses on the effects of edge cracks on the TMI of SC thin films aiming to demonstrate that structural defects dramatically change the stability of superconductivity and may even dominate the modes of flux dynamics in SC films under certain conditions. Edge cracks sharply decrease the threshold magnetic field for the TMI of SC films. The average magnetization and maximum temperature of the SC films with edge cracks jump more frequently with lower amplitudes compared with its crack-less counterpart. As the crack extends, the threshold magnetic field decreases, the jumping frequency gets lower while the jump magnitude gets larger. When the crack extends long enough, the threshold magnetic field increases to even larger than that of the crack-less one. This result originates from the transition of the TMI triggered from the crack tip to the crack edges. This is further validated by the multifractal analysis of the magnetization jumping signals, which shows that multifractal spectrum of the avalanche process transits from multifractal to monofractal as the crack extends. While the crack is small, the magnetization jumping signals show a multifractal spectrum compared with the crack-less film. It is also found that the magnetization jumping follows a power law with a scaling exponent of approximately 1.9. In addition, the physical mechanisms of the TMI of SC thin films with edge cracks are revealed by the TDGL simulations of the dissipative vortex motion. Three different modes of vortex motion are found with the variation of crack lengths, which reproduce well the macroscopic flux dynamics of the films during the dendritic flux avalanche events. In practical applications of SC films, cracks are unavoidable, especially under extreme working conditions. Based on the findings in this work, we can speculate whether the SC film is cracked through analyzing the flux jumping signals, which may initiate a new method to the health monitoring of SC devices. The phase diagram, as well as the threshold field of the TMI, presented in this work may provide guidance to the design and stable operation of SC thin film devices.

## METHODS

### Macroscopic dendritic flux avalanches

The macroscopic flux dynamics of the SC film is characterized by Maxwell's equations [1]:


(1a) \begin{eqnarray*} \nabla \times {\boldsymbol{B}} = {\mu }_0{\boldsymbol{j}}, \end{eqnarray*}

(1b) \begin{eqnarray*} \nabla \cdot {\boldsymbol{B\ }} = {\rm{\ }}0, \end{eqnarray*}

(1c) \begin{eqnarray*} \nabla \times {\boldsymbol{E\ }} = {\rm{\ }} - \frac{{\partial {\boldsymbol{B}}}}{{\partial t}}, \end{eqnarray*}

(1d) \begin{eqnarray*} \nabla \cdot {\boldsymbol{j\ }} = {\rm{\ }}0, \end{eqnarray*}

which should be supplemented by the material constitutive law${\boldsymbol{\ E}} = {\rho }_f( j ){\boldsymbol{j}}$. The heat diffusion process is governed by the thermal diffusion equation, which can be expressed as


(2) \begin{eqnarray*} {C}_p\frac{{\partial T}}{{\partial t}} = \nabla \cdot \kappa \nabla T - \frac{h}{d}\left( {T - {T}_0} \right) + {\boldsymbol{E}} \cdot {\boldsymbol{J}}\!. \end{eqnarray*}

The dendritic flux avalanches in the SC film are simulated by solving the coupled electrodynamics equations and the heat diffusion equation using the fast Fourier transform based iteration scheme [35,38]. See the Supplementary method for details of the numerical scheme and the material parameters adopted.

### Mesoscopic dissipative vortex dynamics [46]

To reveal the physical mechanism of thermomagnetic instabilities, the TDGL equations are solved in combination with the heat diffusion equation to depict the dissipative vortex motion in SC films [47,48]. The dissipated energy due to the induced electric field and the relaxation of the order parameter $\psi $ is taken as derived in Ref. [49]. The coupled TDGL equations and the dimensionless heat diffusion equation are solved using the finite element method. After solving the coupled TDGL equations and the heat diffusion equation, trajectories of the vortices can be captured by the variable $S( {\tilde{x},\tilde{y}} )$ defined as the root mean square of the rate of changes in the local Cooper pair density [50]. Detailed information on the coupled TDGL equations and the heat diffusion equation, as well as material parameters, can be found in the Supplementary method.

### Data analysis procedures

#### Power spectrum estimation

The Welch's method (or the periodogram method) [44] is adopted to estimate the power spectral density of the time series $\{ {X( n ):n\ = \ 0, \ldots ,N - 1} \}$ obtained from flux avalanche simulations. In Welch's method, the time series is divided into ${{M}}$ overlapping segments $\{ {{X}_j( n ) = X( {n + jD} ),n\ = \ 0, \ldots ,N - 1; } j =$${0, \ldots ,M - 1} \}$ with a hop size of *D* between adjacent segments. Each segment is windowed with a Hamming window: ${{W}}( {{n}} ) = 0.54 - 0.46{\rm{\ cos}}( {2\pi n/( {L - 1} )} ),\ 0 \le n \le {\rm{L}} - 1$. Then, the modified periodogram of the *j*-th segment is given by


(3) \begin{eqnarray*} {\tilde{X}}_j\!\left( {f,N} \right) &=& \frac{{\Delta t}}{N}{\left| {\mathop \sum \limits_{n\! =\! {\rm{\ }}0}^{N - 1} W\!\left( n \right){X}_j\! \left( n \right){e}^{ - j2\pi f\Delta tn}} \right|}^2, \\ && -\, \frac{1}{2}{{\Delta t}} < {{f}} \le \frac{1}{2}{{\Delta t}}, \end{eqnarray*}

where $\Delta t$ is the sampling interval. Eventually, the Welch estimate of the PSD is given by


(4) \begin{eqnarray*} {\tilde{S}}_X\! \left( f \right) = \frac{1}{M}\mathop \sum \limits_{j\! =\! {\rm{\ }}0}^{M - 1} {\tilde{X}}_j\left( {f,N} \right)\!. \end{eqnarray*}

In this work, the time series is divided into the longest possible segments but do not exceed 8 segments with 50% overlap.

#### Multifractal formalism

The wavelet leader based multifractal analysis [45] is used to characterize the variability of the flux jumping signals with a collection of scaling exponents. For the signal ${{X}}( {{t}} )$ to be analyzed, its regularity around time ${{{t}}}_0$ is measured locally by the Hölder exponent ${{h}}( {{{{t}}}_0} )$, defined as the largest ${{\alpha }} > 0$, such that there exists a constant ${{C}} > 0$ and a polynomial ${{{P}}}_{{{t}}0}$ of degree less than ${\rm{\alpha }}$, such that $| {{{X}}( {{t}} ) - {{{P}}}_{{{t}}0}( t )} | \le {{C}}{| {t - {t}_0} |}^\alpha $ in a neighborhood of ${{{t}}}_0$. The variability of the signal is described by the multifractal spectrum ${{D}}( {{h}} )$ which is defined as the Hausdorff dimensions of the set of points ${{{t}}}_{{i}}$ where ${{h}}( {{{{t}}}_{{i}}} ) = {{h}}$. Scaling properties of the signal ${{X}}( {{t}} )$ is determined by assuming and checking that its structural functions ${{S}}( {{{q}},{{\ }}a} )$ behave as power laws of the scale *a*, for a given range of $a \in [ {{a}_{{m}},{a}_{{M}}} ]$ and a given range of statistical moments ${{q}}$:


(5) \begin{eqnarray*} {{S}}\left( {{{q}},a} \right) = \frac{1}{{{n}_a}}\mathop \sum \limits_{k\! =\! {\rm{\ }}1}^{{n}_a} {\left| {{T}_X\! \left( {a,k} \right)} \right|}^q \cong {a}^{\zeta \left( q \right)}, \end{eqnarray*}

where ${T}_X( {a,k} )$ are the wavelet leaders determined by the wavelet coefficients, ${n}_a$ is the number of these coefficients at the scale *a*, $\zeta ( q )$ is the scaling exponent. When $\zeta ( q )$ is a linear function of *q*, the signal is monofractal. While it deviates from being linear, the signal is multifractal.

## Supplementary Material

nwad052_Supplemental_File
