# Supplementary material for: Effects of edge cracks on the thermomagnetic instabilities of type-II superconducting thin films
Source: Natl Sci Rev. 2023 Feb 28;10(3):nwad052. doi: 10.1093/nsr/nwad052 (PMC10062698; doi:10.1093/nsr/nwad052)
Supplement: nwad052_Supplemental_File [file nwad052_supplemental_file.docx]

Supplementary information for

**Effects of Edge Cracks on the Thermomagnetic Instabilities of Type-II Superconducting Thin Films**

**Ze Jing***

Institute of Extreme Mechanics and School of Aeronautics, Northwestern Polytechnical University, Xi'an 710072, People's Republic of China

*Corresponding Author Email: zejing@nwpu.edu.cn

**Supplementary information**

**This file includes:**

Supplementary Methods

Supplementary References

**Supplementary Methods:**

***Macroscopic Dendritic Flux Avalanches***

The macroscopic flux dynamics of the SC film is characterized by the Maxwell’s equations [1,2]:

$\boldsymbol{\nabla}\times\boldsymbol{B}=\mu_{0}\boldsymbol{j}$ (1a)$\boldsymbol{\nabla}\cdot\boldsymbol{B}=0$ (1b)$\boldsymbol{\nabla\times}\boldsymbol{E}=-\frac{\partial\boldsymbol{B}}{\partial t}$ (1c)$\boldsymbol{\nabla}\cdot\boldsymbol{j}=0$ (1d)

which should be supplemented by the material constitutive law$\boldsymbol{E}=\rho_{f}(j)\boldsymbol{j}$. The heat diffusion process is governed by the thermal diffusion equation, which can be expressed as

$C_{p}\frac{\partial T}{\partial t}=\boldsymbol{\nabla}\cdot\kappa\boldsymbol{\nabla}T-\frac{h}{d}\left( T-T_{0} \right)+\boldsymbol{E\cdot J}$ (2)

where $C_{p}$ is the specific heat, $\kappa$ is the thermal conductivity, $h$ is the heat transfer coefficient, and $\kappa$, $C_{p}$, and $h$ are all temperature dependent.

In the fast fourier transform (FFT) based numerical scheme, Eqs. (1) are solved using the integral method [3,4]. The sheet current density is derived as a stream function of local magnetization $g=g(x,y )$,

$J=\nabla\times zg$ (3)

where $z$ is the unit vector perpendicular to the film-plane. The total magnetic field $\boldsymbol{H}_{z}$ is the sum of the SC current induced field $\boldsymbol{H}_{S}$ and the applied field $\boldsymbol{H}_{a}$, i.e.

$\boldsymbol{H}_{z}=\boldsymbol{H}_{a}+\boldsymbol{H}_{S}(x,y,t)$ (4)

where $\boldsymbol{H}_{\boldsymbol{S}}(x,y,t)$ is the SC current induced field. According to the Biot-Savart’s law, $\boldsymbol{H}_{S}$ can be written as $\boldsymbol{H}_{S}(x,y,t)=\int_{S} d^{2}r'Q(\boldsymbol{r},\boldsymbol{r'})g(\boldsymbol{r}')$, in which $Q(\boldsymbol{r},\boldsymbol{r}')$ denotes the 2-dimensional green’s function satisfying $\mathcal{F}\left[ Q \right]=\left\lceil\mathbf{k} \right\rceil/2$ with $\mathcal{F}$ denotes the Fourier transform, $\mathbf{r}=(x,y)$ and $\mathbf{k}$ the in-plane wave vector in the Fourier space [2]. In the SC film, time derivative of the total magnetic field, $d\boldsymbol{H}_{z}/dt$, can be obtained from the constitutive law along with the Faraday’s law $(1c)$,

$\frac{\partial H_{z}}{\partial t}=\nabla\cdot\left( \rho_{f}\nabla g \right)/\mu_{0}d$ (5)

Applying Fourier transform on both side of Eq. (4) and using the convolution theorem, it can be derived that [2]

$\frac{\partial g}{\partial t}=\mathcal{F}^{-1}\left[ \frac{2}{k}\mathcal{F}\left[ \nabla\cdot\left( \rho_{f}\nabla g \right)/\mu_{0}d-\frac{\partial H_{a}}{\partial t} \right] \right]$ (6)

Eq. (6) is solved with the FFT based iteration scheme [1], and by coupling with Eq. (2) and, the flux avalanches in SC film can be tracked. In this work, the method of lines is used to solve the coupled electromagnetic-thermal equations. The simulation domain is discretized into equidistant grids, so that the partial differential equations are reduced to a system of ordinary differential equations (ODEs). The resulting ODEs are integrated in time by the Runge-Kutta scheme.

***Coupled TDGL Equations and Heat Diffusion Equation***

In dimensionless form, the TDGL equations can be reduced to the following coupled nonlinear partial differential equations [4]:

$\frac{u}{\sqrt{1+\gamma^{2}\left| \psi\right|^{2}}}\left( \frac{\partial}{\partial\tilde{t}}+\frac{\gamma^{2}}{2}\frac{\partial\left| \psi\right|^{2}}{\partial\tilde{t}} \right)\psi=-\left( -i\tilde{\nabla}-\tilde{A} \right)^{2}\psi+\psi\left( 1-\tilde{T}-\left| \psi\right|^{2} \right)$ (7)

$\kappa^{2}\tilde{\boldsymbol{\nabla}}\times\tilde{\boldsymbol{\nabla}}\times\tilde{\boldsymbol{A}}={\tilde{\boldsymbol{J}}}_{s}-\left( \frac{\partial\tilde{\boldsymbol{A}}}{\partial\tilde{t}}+\tilde{\boldsymbol{\nabla}}\varphi\right)$ in $\Omega$ (8)

in which $\psi$ is the order parameter, and $\tilde{\boldsymbol{A}}$ is the vector potential describing the magnetic field as $\tilde{\boldsymbol{B}}\boldsymbol{=}\tilde{\boldsymbol{\nabla}}\times\tilde{\boldsymbol{A}}$, $\tilde{T}$ is the temperature, $\kappa$ is the Ginzburg-Landau parameter, and $u$ and $\gamma$ are parameters related to the relaxation of order parameter. ${\tilde{\boldsymbol{J}}}_{s}$ is the superconducting current density defined as ${\tilde{\boldsymbol{J}}}_{s}=\left( \bar{\psi}\tilde{\boldsymbol{\nabla}}\psi-\psi\tilde{\boldsymbol{\nabla}}\bar{\psi} \right)/{2i}-\tilde{\boldsymbol{A}}\left| \psi\right|^{2}$, in which $\bar{\psi}$ is the conjugate of $\psi$. The dimensionless units are chosen as follows: lengths are scaled in units of the coherence length $\xi(0)$, time in $t_{GL}(0)=\pi\hbar/8k_{b}T_{c}u$, magnetic field in the upper critical magnetic field $H_{c2}(0)$, the vector potential $\tilde{\boldsymbol{A}}$ in $H_{c2}(0)\xi(0)$, the temperature in $T_{c}$, and $\varphi$ in $\hbar/2et_{GL}(0)$.

The coupling between vortex motion and energy dissipation plays a crucial role in the thermomagnetic instability of the superconductors. The dissipated energy, $\tilde{Q}$, in units of $Q_{0}=H_{c2}^{2}\left( 0 \right)/\left[ 8\pi\kappa^{2}t_{GL}\left( 0 \right) \right]$, can be given by [5, 6]

$\tilde{Q}=\left( \frac{\partial\tilde{A}}{\partial\tilde{t}} \right)^{2}+\frac{u}{\sqrt{1+\gamma^{2}\left| \psi\right|^{2}}}\left[ \left( \left| \frac{\partial\psi}{\partial\tilde{t}} \right| \right)^{2}+\frac{\gamma^{2}}{4}\left( \frac{\partial\left| \psi\right|^{2}}{\partial\tilde{t}} \right)^{2} \right]$ (9)

in which the first term is the heat dissipated by the induced electric field, and the second term is the dissipation due to the relaxation of the order parameter. Thus, the dimensionless heat diffusion equation can be written as

$C_{eff}\frac{\partial\tilde{T}}{\partial\tilde{t}}=\tilde{\nabla}\cdot\left( \kappa_{eff}\tilde{\nabla}\tilde{T} \right)+\tilde{Q}-h_{eff}\left( \tilde{T}-\tilde{T}_{0} \right)$ (10)

where $\tilde{T}_{0}$ is the ambient temperature, and$C_{eff}$, $K_{eff}$ and $h_{eff}$ are the effective heat capacity, thermal conductivity and heat transfer coefficient, respectively.

Trajectories of the vortices can be captured by the variable $S(\tilde{x},\tilde{y})$ defined as the root mean square of the rate of changes in the local Cooper pair density [7,8]:

$S(\tilde{x},\tilde{y})=\sqrt{\frac{1}{\tilde{t}_{2}-\tilde{t}_{1}}\int_{\tilde{t}_{1}}^{\tilde{t}_{2}} \left( \frac{\partial\left| \psi(\tilde{x},\tilde{y}) \right|^{2}}{\partial\tilde{t}} \right)^{2}d\tilde{t}}$ (11)

where$\tilde{t}_{2}-\tilde{t}_{1}$ is the time interval during which the vortex trajectories are tracked. It is obvious that $S$ at a given point $\left( \tilde{x},\tilde{y} \right)$ increases with vortices passing through.

In the simulation of the dissipative vortex motion, the coupled TDGL equations and the dimensionless heat diffusion equation are solved using the finite element method. The simulation domain is discretized with triangular meshes with the maximum size $l_{max}=0.5$, and the time is integrated with the Backward differentiation formulas (BDF) scheme. To guarantee the accuracy of the numerical scheme, the relative tolerance of each iteration is set to $\varepsilon\leq1\times{10}^{-5}$. The material parameters are taken as$\kappa=10$, $u=5.79$, $\gamma=10$, $C_{eff}=0.65$, $K_{eff}=0.06$, and $h_{eff}=2\times{10}^{-3}$.

**Supplementary References**

1. Tinkham M. Introduction to Superconductivity. 2nd ed. New York: Dover Publications, 2004

2. Vestgården J, Mikheenko P, Galperin Y *et al.* Nonlocal electrodynamics of normal and superconducting films. *New J Phys* 2013; **15**:093001.

3. Brandt EH. Electric field in superconductors with rectangular cross section. *Phys Rev B* 1995; 52:15442–57.

4. Schuster T, Kuhn H & Brandt EH. Flux penetration into flat superconductors of arbitrary shape: Patterns of magnetic and electric fields and current. *Phys Rev B* 1996; 54:3514-24.

5. Machida M, Kaburaki H. Direct simulation of the time-dependent Ginzburg-Landau equation for type-II superconducting thin film: Vortex dynamics and *V* - *I* characteristics. *Phys Rev Lett* 1993; **71**:3206–9.

6. Vodolazov DY, Peeters F M, Morelle M et al. Masking effect of heat dissipation on the current-voltage characteristics of a mesoscopic superconducting sample with leads. *Phys Rev B* 2005; 71:184502.

7. Gladilin VN, Tempere J, Devreese JT *et al.* Vortex-antivortex pair generation by an in-plane magnetic dipole on a superconducting film. *Phys Rev B* 2009;**80**:054503.

8. Brisbois J, Gladilin VN, Tempere J *et al.* Flux penetration in a superconducting film partially capped with a conducting layer. *Phys Rev B* 2017;**95**:094506.
